# Recurrent emergence of *Klebsiella pneumoniae* carbapenem resistance mediated by an inhibitory *ompK36* mRNA secondary structure

**DOI:** 10.1073/pnas.2203593119

**Published:** 2022-09-12

**Authors:** Joshua L. C. Wong, Sophia David, Julia Sanchez-Garrido, Jia Z. Woo, Wen Wen Low, Fabio Morecchiato, Tommaso Giani, Gian Maria Rossolini, Konstantinos Beis, Stephen J. Brett, Abigail Clements, David M. Aanensen, Silvi Rouskin, Gad Frankel

**Affiliations:** ^a^Centre for Molecular Bacteriology and Infection, Department of Life Sciences, Imperial College London, London SW7 2AZ, United Kingdom;; ^b^Department of Surgery and Cancer, Section of Anaesthetics, Pain Medicine and Intensive Care, Imperial College London, London SW7 2AZ, United Kingdom;; ^c^Centre for Genomic Pathogen Surveillance, Big Data Institute, University of Oxford, Oxford OX3 7LF, United Kingdom;; ^d^Department of Microbiology, Harvard Medical School, Boston, MA 02115;; ^e^Department of Experimental and Clinical Medicine, University of Florence, Florence 50134, Italy;; ^f^Clinical Microbiology and Virology Unit, Careggi University Hospital, Florence 50134, Italy;; ^g^Department of Life Sciences, Imperial College London, London SW7 2AZ, United Kingdom;; ^h^Rutherford Appleton Laboratory, Research Complex at Harwell, Didcot, Oxfordshire OX11 0FA, United Kingdom

**Keywords:** Antibiotic resistance, *Klebsiella pneumoniae*, Carbapenems, Outer membrane porins, Synonymous mutations

## Abstract

Outer membrane porins in Gram-negative bacteria facilitate antibiotic influx. In *Klebsiella pneumoniae*, modifications in the porin OmpK36 are implicated in increasing resistance to carbapenems. An analysis of large *K. pneumoniae* genome collections, encompassing major healthcare-associated clones, revealed the recurrent emergence of a synonymous cytosine-to-thymine transition at position 25 (25c > t) in *ompK36.* We show that the 25c > t transition increases carbapenem resistance through depletion of OmpK36 from the outer membrane. The mutation attenuates *K. pneumoniae* in a murine pneumonia model, which accounts for its limited clonal expansion observed by phylogenetic analysis. However, in the context of carbapenem treatment, the 25c > t transition tips the balance toward treatment failure, thus accounting for its recurrent emergence. Mechanistically, the 25c > t transition mediates an intramolecular messenger RNA (mRNA) interaction between a uracil encoded by 25t and the first adenine within the Shine–Dalgarno sequence. This specific interaction leads to the formation of an RNA stem structure, which obscures the ribosomal binding site thus disrupting translation. While mutations reducing OmpK36 expression via transcriptional silencing are known, we uniquely demonstrate the repeated selection of a synonymous *ompK36* mutation mediating translational suppression in response to antibiotic pressure.

## Significance

Carbapenem-resistant *Klebsiella pneumoniae* represents an urgent threat to human health. Together with carbapenemase-mediated hydrolysis, mutations in the outer membrane porin OmpK36 have evolved to limit carbapenem influx. Analysis of the *ompK36* gene from high-risk *K. pneumoniae* sequence types revealed the repeated emergence of an identical 5′ synonymous mutation. Whilst synonymous mutations are usually considered silent, we show that it reduces OmpK36 translation by inducing the formation of a messenger RNA secondary structure that obstructs the ribosomal binding site. While OmpK36 depletion attenuates virulence in a mouse lung infection model, it tips the balance towards antibiotic therapy failure. These results show mechanistically how the de novo emergence of a synonymous mutation contributes to last line antimicrobial resistance.

Classical outer membrane (OM) porins in Gram-negative bacteria enable nonspecific bidirectional diffusion between the periplasm and extracellular environment (1). This has been exploited in antibacterial chemotherapy, as porins act as the key entry point for clinically important classes of antibiotics across the otherwise impermeable OM. However, porin modifications that restrict antibiotic entry have evolved in response to this selective pressure (2), contributing to the rising global burden of resistant bacterial infections, especially among species of *Enterobacteriaceae* (3–6).

*Klebsiella pneumoniae* is one of the most clinically significant members of the *Enterobacteriaceae* family and a leading cause of healthcare-associated infections worldwide (7, 8). The majority of resistant *K. pneumoniae* infections are caused by “high-risk” clonal lineages, including sequence type 258 (ST258) and ST512, which form a dominant clone associated with the *K. pneumoniae* carbapenemase (KPC) gene (9). Together with plasmid-encoded carbapenemases, modifications to the major OM porins OmpK35 and OmpK36 play a critical role in mediating *K. pneumoniae* resistance to carbapenems, a class of antibiotics that is vital for the treatment of severe infections. Resistance-associated modifications broadly fall into those that structurally alter the porin channel and those that abolish or reduce OmpK36 expression.

Structural alterations in OmpK36 are mediated by amino acid insertions into a region of the porin called loop 3 (L3) (10). These insertions narrow the luminal diameter and restrict substrate diffusion, including antibiotics (5, 6). L3 insertions are relatively prevalent among clinical *K. pneumoniae* isolates, having been found among 12.3% (192/1,557) of isolates from a diverse public genome collection (6). We previously showed that the most common L3 insertion, a di-amino acid insertion (glycine-aspartate, GD), results in a 16-fold increase in the minimum inhibitory concentration (MIC) to meropenem (5).

In addition to structural modifications, carbapenem resistance is also achieved by absent or reduced OmpK36 expression. Absent expression, which can be achieved by gene truncation, results in high levels of resistance but comes at a significant in vivo fitness cost (5, 6, 11). Reduced OmpK36 expression has been reported to occur by multiple mechanisms that all result from transcriptional silencing, including *ompK36* promoter disruption by insertion sequence elements (12), loss-of-function mutations in *kvrA* (a transcriptional repressor that controls capsule production) (13), and mutations in *hfq* (a regulatory RNA binding protein) (14). However, the effects on virulence resulting from reduced OmpK36 expression are poorly understood. Moreover, the prevalence and clinical significance of these mechanisms remain unknown as the mutations identified to date were either restricted to clinical isolates from a single center (promoter insertion) or evolved during in vitro selection or genetic deletion experiments (*kvrA* and *hfq*).

A growing body of evidence suggests that synonymous mutations, which do not alter encoded amino acids and are often assumed to be neutral, could have beneficial or detrimental bacterial fitness outcomes; however, the underlying mechanism in most cases is not well understood (15, 16). The evidence for this is largely based on results from in vitro evolution studies. These have shown that synonymous mutations can affect transcription (17), translation elongation (18), and protein folding and RNA stability/structure (15). While they represent diverse mechanisms, it is known that the 5′ termini of almost all bacterial messenger RNA (mRNA) transcripts display a reduction in predicted secondary structure (19).

For example, laboratory evolution experiments utilizing *Pseudomonas fluorescens*, grown with glucose as the sole carbon source, identified the selection of two beneficial synonymous mutations in *gtsB*, encoding a permease subunit of an ABC glucose transporter (16). A follow-up study suggested that the fitness benefit arose from the binding of transcription factors to promoter-like sequences in *gtsB*, followed by enhanced transcription of downstream genes (17). In addition, during the in vitro evolution of *Salmonella enterica* serovar Typhimurium in similar conditions, a synonymous 5′ terminal mutation that increased growth rate was identified within *proA* (15). Computational analyses in this region suggested that the mutation altered the 5′ mRNA structure and increased the efficiency of translation initiation. However, while in vitro evolution in minimal media revealed that synonymous mutations are not all silent, very little is known about their natural emergence in clinically important populations of bacteria and their impact on in vivo fitness.

Here, we describe a carbapenem resistance mechanism that has repeatedly evolved in clinical *K. pneumoniae* isolates to posttranscriptionally alter OmpK36 abundance via synonymous mutations in the open reading frame (ORF). Our study spans the identification of one such key mutation (25c > t) in *ompK36* through large-scale bioinformatic approaches, the assessment of its effects on carbapenem susceptibility and virulence using murine pneumonia models, and finally the determination of the precise molecular mechanism linking synonymous single nucleotide polymorphisms (SNPs) with protein abundance. In particular, we show that the 25c > t mutation results in the formation of a stem structure in the *ompK36* mRNA that obscures the Shine–Dalgarno sequence (SDS), reduces OmpK36 abundance in the OM, and increases carbapenem resistance. This work provides an example of the functional use of these inhibitory mRNA structures in regulating protein expression, antibiotic resistance, and bacterial adaptation in a clinically important human pathogen.

## Results

### Recurrent Emergence of a Synonymous 25c > t *ompK36* Mutation.

**Fig. 1.**
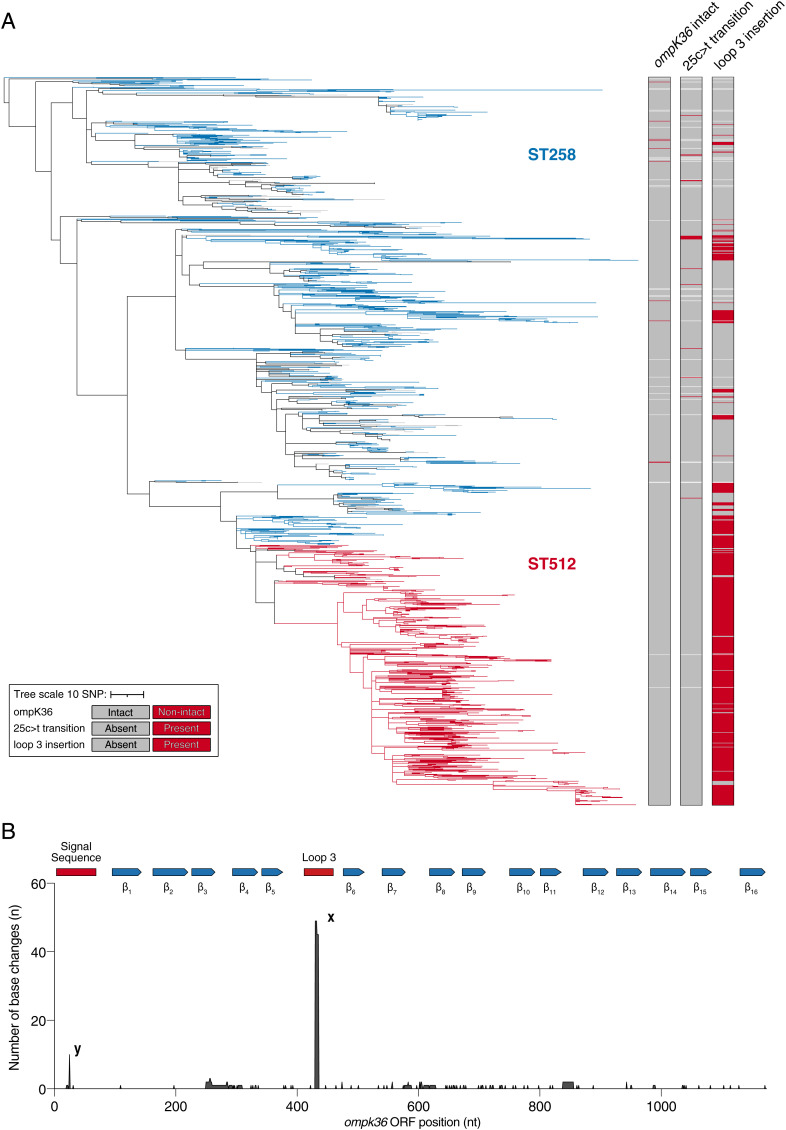
Frequency and phylogenetic distribution of *ompK36* variants in a curated collection of ST258/512 genomes. (*A*) Phylogenetic tree of 1,450 *K. pneumoniae* ST258/512 isolates constructed using vertically inherited SNPs. Branches are colored blue if all descendent taxa belong to ST258, red if they belong to ST512, gray if they belong to other closely related derivatives of these STs, or black if they belong to one or more of these different categories. Data columns (L to R) show *ompK36* (intact/nonintact), *ompK36* 25c > t transition (present/absent), and *ompK36* L3 insertion (present/absent) in each genome. Isolates are marked white if *ompK36* could not be unambiguously identified in the genome (columns 1 to 3) or if *ompK36* was nonintact (columns 2 and 3). The scale represents the number of SNPs per variable site. A similar visualization is available online at https://microreact.org/project/1vWbaqARPRNc55n4yfdLyQ-ompk36#wksn-figure-1a-wong-et-al-2022. (*B*) The number of changes (including SNPs, insertions, and deletions) at each position in *ompK36* detected across the ST258/512 tree. The protein structure motifs (SS, beta strands 1 to 16, and L3) are plotted on the top of the sequence as a reference. The labeled peaks represent insertions/reversions within the L3 (x) and the 25c > t mutation found within the SS (y).

**Fig. 2.**
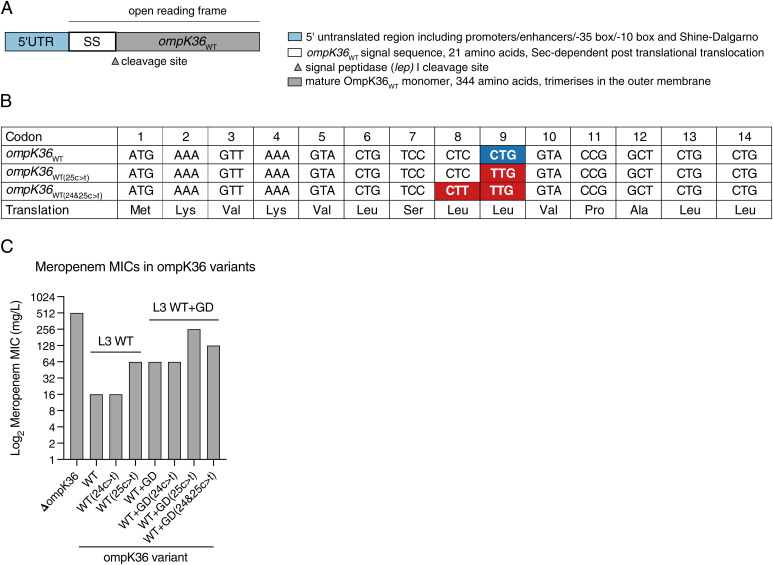
*ompK36*_WT(25c > t)_ leads to increased meropenem MIC and reduced OmpK36 abundance. (*A*) Schematic of the *ompK36* locus. (*B*) The synonymous 25c > t mutation occurs at codon position 9 (Leu9) resulting in a change of the codon from CTG (*ompK36*_WT_) to TTG (*ompK36*_WT(25c > t)_). The 24c > t mutation occurs in position 8 (Leu8) resulting in a synonymous CTC to CTT codon switch. (*C*) The 25c > t transition increases the meropenem resistance achieved on both a WT and WT+GD *ompK36* background. The additional 24c > t (WT+GD(24&25c > t)) mutation partially reverses the resistance achieved in the 25c > t transition. The 24c > t mutations alone do not influence the meropenem MIC. All strains harbor a pKpQIL-like plasmid expressing KPC-2 and have *ompK35* deleted. Resistance values represent broth MICs; graphs show a median of 2 (in both 24c > t single mutants) or 3 biological repeats (rest of the mutants). The MIC was identical in all assays, and therefore, no error bars are shown.

We curated and analyzed a collection of 1,450 public *K. pneumoniae* genomes belonging to the major healthcare-associated clone composed of ST258 and ST512 and other closely related derivatives (https://microreact.org/project/1vWbaqARPRNc55n4yfdLyQ-ompk36; Dataset S1). The collection comprises isolates gathered between 2003 and 2018, largely in the Americas, Europe, and the Middle East, where an extensive spread of ST258/512 in healthcare institutions has been reported (19–23). We unambiguously identified the *ompK36* gene in 98.1% (1,422/1,450) of the genomes. Among these, the gene was intact in 99.3% (1,412/1,422). Using the intact *ompK36* sequences, we inferred how each position in this gene has evolved across the ST258/512 population. First, we constructed a phylogeny of the collection using all vertically inherited SNPs from a core genome alignment, with recombined regions excluded, to provide an accurate representation of the ancestral relationships between isolates (Fig. 1*A*). We then mapped the variation in *ompK36* onto this phylogeny and predicted the ancestral states of each position in the gene across the tree using a maximum parsimony approach. While 83.4% of nucleotide positions are entirely conserved across *ompK36*, we found two regions with an increased number of base changes (Fig. 1*B*). As anticipated, the largest number of changes occurred in the L3 region comprising amino acid insertions (GD, threonine-aspartate, aspartate, and asparagine) (24 occurrences) as well as their reversions (i.e., deletions) (25 occurrences). Moreover, we observed 10 base changes at position 25, with all consisting of a synonymous c > t transition (CTG (leucine) > TTG (leucine)). Despite this mutation maintaining the identical amino acid translation in the Sec-dependent signal sequence (SS, Fig. 2*A* and *B*), its high frequency of emergence suggests that it has undergone positive selection. The majority of isolates with the 25c > t mutation are sporadically distributed across the core genome phylogeny and occur as singletons or clusters of only two isolates (11/18) (Fig. 1*A*). The remaining seven isolates, collected between 2009 and 2013 from multiple healthcare institutions in the United States, form a monophyletic cluster, which is indicative of a clonal expansion. We noted that these seven isolates encode an additional synonymous 24c > t mutation in the preceding codon ((CTC (leucine) > CTT (leucine)) (Fig. 2*B*).

We next searched for the 25c > t mutation in a large, geographically diverse collection of 16,086 *K. pneumoniae* genomes (https://pathogen.watch/genomes/all?genusId=570&speciesId=573; Dataset S2) to establish its wider prevalence among sequenced isolates and presence in other (i.e., non-ST258/512) clonal lineages. Among the 14,888 isolates encoding an intact *ompK36* gene, we identified the 25c > t mutation in 2.5% (376/14,888), which includes isolates from 39 STs and 25 countries. Over half (51.3%, 193/376) belong to ST258, while many also belong to other globally important multidrug-resistant clones (e.g., ST15, 19.9%; ST13, 4.5%; ST11, 2.7%). We observed the 24c > t mutation in 1.1% (161/14,888) of genomes from this collection, including four STs (albeit mostly ST258 [158/161]) and only ever in combination with the 25c > t mutation. Moreover, we also noted that 93.2% (150/161) of *ompK36* sequences containing the 24&25c > t mutations had L3 insertions compared to only 1.9% (4/215) of those with 25c > t only.

### The 25c > t Mutation Increases Meropenem Resistance.

We investigated the effect of the 24c > t and 25c > t *ompK36* mutations on meropenem resistance in a background resembling that of KPC-producing *K. pneumoniae* strains of the ST258/512 lineage, using derivatives of the laboratory *K. pneumoniae* strain ICC8001 (5). We refer to the genes in this strain as wild type (WT), in line with previous work (5, 24). To that end, we deleted the gene encoding *ompK35* (as this is truncated in 99.8% of the ST258/512 genome collection) and introduced a pKpQIL-like plasmid encoding the KPC-2 carbapenemase (24) (Tables 1 and 2). We then superimposed 24c > t and 25c > t mutations into the chromosomal *ompK36* locus (Fig. 2*A*), which correspond to codons 8 and 9 of the SS.

**Table 1.** *ompK36* variants used in this study

*ompK36* variants	Nucleotide at position 24	Nucleotide at position 25	L3 insertion
ompK36_WT_	c	c	Absent
ompK36_WT(25c > t)_	c	t	Absent
ompK36_WT+GD_	c	c	GD
ompK36_WT+GD(25c > t)_	c	t	GD
ompK36_WT+GD(24&25c > t)_	t	t	GD

**Table 2.** *K. pneumoniae* strains used in this study

Strains	ompK35	ompK36	Carbapenemase*
KPΔ36	Δ	Δ	+/− KPC-2
KP36_WT_ (+/− KPC-2)	Δ	ompK36_WT_	+/− KPC-2
KP36_WT(25c > t)_ (+/− KPC-2)	Δ	ompK36_WT(25c > t)_	+/− KPC-2

^*^KPC-2 on pKpQIL-like plasmid.

The presence of the 25c > t mutation was associated with a fourfold increment of the meropenem MIC in the presence of either a WT OmpK36 (OmpK36_WT_) (64 vs. 16 mg/L) or OmpK36 carrying an L3 GD insertion (OmpK36_WT+GD_) (256 vs. 64 mg/L). In the latter, the meropenem MIC almost equaled that of Δ*ompK36* (512 mg/L) (Fig. 2*C*). In contrast, the presence of the 24c > t mutation alone (which is not observed among the *K. pneumoniae* genomes analyzed) had no effect on the meropenem MIC in the presence of either OmpK36_WT_ or OmpK36_WT+GD_ (Fig. 2*C*).

The introduction of a double 24c > t&25c > t mutation alone was not tolerated on a WT *ompK36* background in the ICC8001 genome. However, this double mutation could be introduced in the context of OmpK36_WT+GD_. Here, the increment in meropenem MIC was lower than that observed with the single 25c > t mutant (twofold rather than fourfold) (Fig. 2*C*). Thus, when encountered in the context of an adjacent 25c > t mutation, the 24c > t mutation appeared to partially counteract the effect of the former, reducing the final meropenem resistance level. While we could not explain the inability to generate the double mutant on a WT *ompK36* background, this observation nevertheless reflected the association of the 24&25c > t double mutation with L3 insertions among clinical genomes.

### *ompK36*_WT(25c > t)_ Attenuates Virulence but Tips the Balance toward Antibiotic Therapy Failure.

**Fig. 3.**
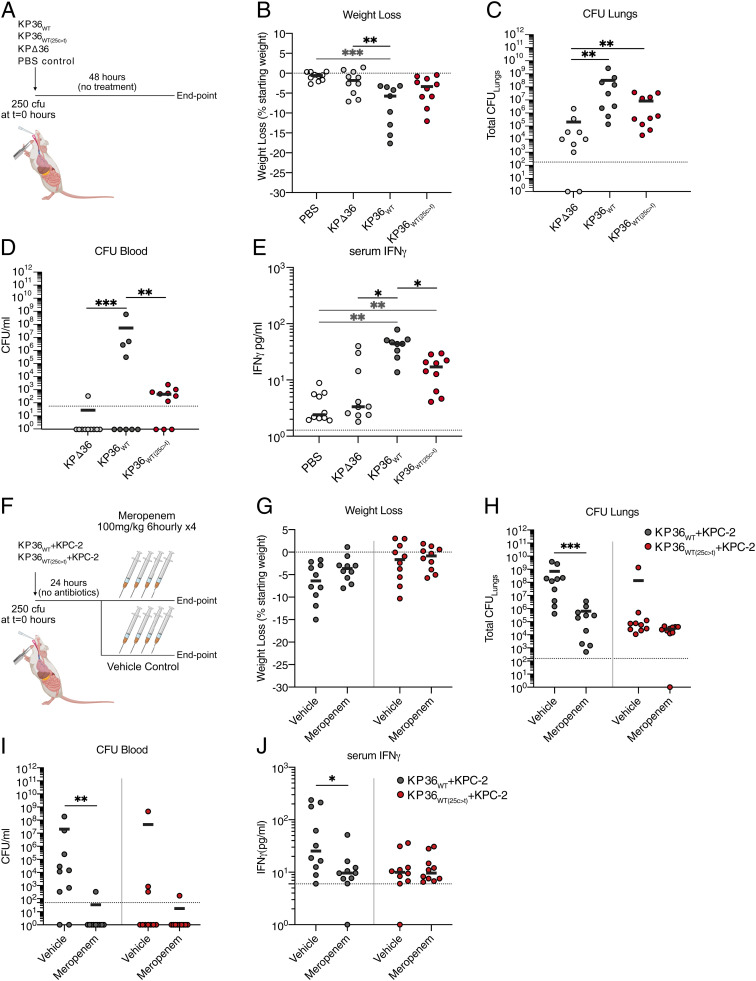
The 25c > t mutation has a fitness cost in vivo but is advantageous in the context of meropenem therapy. (*A*–*E*) Mice were infected by intubation and administration of 250 CFUs of KP36_WT_, KP36_WT(25c > t)_, or KPΔ36 *K. pneumoniae* strains; mock infection with PBS was used as a control. A schematic of the infection protocol is outlined in *A*. (*B*) Animals infected with KP36_WT_ show greater weight loss after 48 h compared to mock-infected animals and those infected with KPΔ36. Weight loss in animals infected with KP36_WT(25c > t)_ is not significantly different to that of the PBS or KPΔ36 groups. (*C* and *D*) Enumeration of CFUs in lungs and blood, collected at 48 h postinfection, reveals that infection with KPΔ36 does not result in high lung bacterial burdens and bacteremia. Infection with KP36_WT_ results in high pulmonary bacterial burdens I and bacteremia (*D*), while infection with KP36_WT(25c > t)_ results in a trend toward lower pulmonary bacterial counts and reduced levels of bacteria in the blood. (*E*) Infection with KP36_WT_ or KP36_WT(25c > t)_ leads to increased serum IFNγ compared to mock-infected (PBS) animals. KP36_WT(25c > t)_ infection results in significantly reduced serum IFNγ levels compared to KP36_WT_ infection. (*F*–*J*) Mice were administered with KP36_WT_+KPC-2 or KP36_WT(25c > t)_+KPC-2 strains and subjected to a meropenem dosing regimen to assess the success of antibiotic therapy in *K. pneumoniae*-induced severe pneumonia. A schematic of the infection protocol is outlined in *F*. (*G*) No changes were observed in weight loss between meropenem and vehicle control therapy when experimental pneumonia was induced by either KP36_WT_+KPC-2 or KP36_WT(25c > t)_+KPC-2. (*H* and *I*) CFU enumeration in lungs (*H*) and blood (*I*) shows significant reduction in meropenem-treated animals infected with KP36_WT_+KPC-2 compared to vehicle-treated animals; levels of bacteremia were undetectable in all but one meropenem-treated animal. Meropenem treatment has no impact on lung or blood CFUs in animals infected with KP36_WT(25c > t)_+KPC-2. (*J*) Serum levels of IFNγ are significantly reduced after meropenem treatment in animals infected with KP36_WT_+KPC-2; cytokine levels are not affected by meropenem treatment in animals infected with KP36_WT(25c > t)_+KPC-2. Graphs show median values of 2 biological repeats (4 to 5 mice per group). Statistical significance was determined by one-way ANOVA with Tukey’s multiple comparison posttest. For multiple comparisons, only significant changes are indicated in the figure, where *P < 0.05, **P < 0.01, and ***P < 0.001 in *B*–*E*. All other comparisons were nonsignificant (*P* > 0.05). The statistical significance of the comparisons between vehicle and meropenem-treated animals was determined via a nonparametric Mann-Whitney test. *P < 0.05, **P < 0.01, ***P < 0.001 in *G* to *J*. Dotted lines represent the limit of detection in all graphs.

The general absence of clonal expansion among isolates with the single 25c > t substitution, despite the frequent emergence of this mutation, suggests that it may have a fitness cost that impedes onward transmission. We tested this hypothesis in vivo by infecting mice with 250 colony-forming units (CFUs) of *K. pneumoniae* encoding either *ompK36*_WT_ (KP36_WT_) or *ompK36*_WT(25c > t)_ (KP36_WT(25c > t)_) (Tables 1 and 2). Inoculation with a strain encoding an *ompK36* deletion (KPΔ36) and phosphate-buffered saline (PBS) were used as controls (Fig. 3*A*). At 48 h postinfection, KP36_WT_, but not KP36_WT(25c > t)_, induced significant weight loss compared to uninfected (PBS) mice or mice infected with KPΔ36 (Fig. 3*B*). Bacterial counts in the lungs showed a trend (*P* = 0.0527) pointing toward higher burdens in mice infected with KP36_WT_ than with KP36_WT(25c > t)_, which reached significance in the blood (Fig. 3*C* and *D*). KPΔ36 demonstrated defects in bacterial survival and proliferation in the host, with significantly lower and/or absent burdens in the lungs and blood (Fig. 3*C* and *D*). Consistently, infection with KP36_WT_ triggered significantly higher IFNγ responses compared to infection with KP36_WT(25c > t)_ (Fig. 3*E*). Taken altogether, these findings show that the 25c > t transition attenuates *K. pneumoniae* in vivo with reduced bacteremia, which was mirrored by a diminished host proinflammatory immune response.

We next evaluated if *ompK36*_WT(25c > t)_ provides an advantage compared with *ompK36*_WT_ in the context of meropenem therapy, which would explain the recurrent emergence of this mutation despite its associated fitness cost. We infected mice with either KP36_WT_ or KP36_WT(25c > t)_, both expressing the KPC-2 carbapenemase from a pKpQIL-like plasmid. At 24 h, mice either received meropenem (100-mg/kg dose) or vehicle control (water) by intraperitoneal injection at six hourly intervals for 24 h (Fig. 3*F*). The experiment was stopped 3 h after the last injection. No significant differences in body weight were observed following infection by either strain with or without meropenem therapy (Fig. 3*G*), consistent with fluid resuscitation provided by meropenem or vehicle administration. However, significant reductions in the bacterial burdens in the lungs and blood were seen following meropenem treatment of KP36_WT_+KPC-2 but not KP36_WT(25c > t)_+KPC-2 infection (Fig. 3*H* and *I*). Furthermore, while the level of serum IFNγ was significantly decreased when KP36_WT_+KPC-2 infection was treated with meropenem (Fig. 3*J*), no significant differences were observed between antibiotic or mock-treated KP36_WT(25c > t)_+KPC-2 infected mice. These in vivo results show while the 25c > t mutation in *ompK36* attenuates *K. pneumoniae*, it provides a selective advantage during carbapenem therapy.

### The 25c > t Mutation Reduces OmpK36 Abundance.

**Fig. 4.**
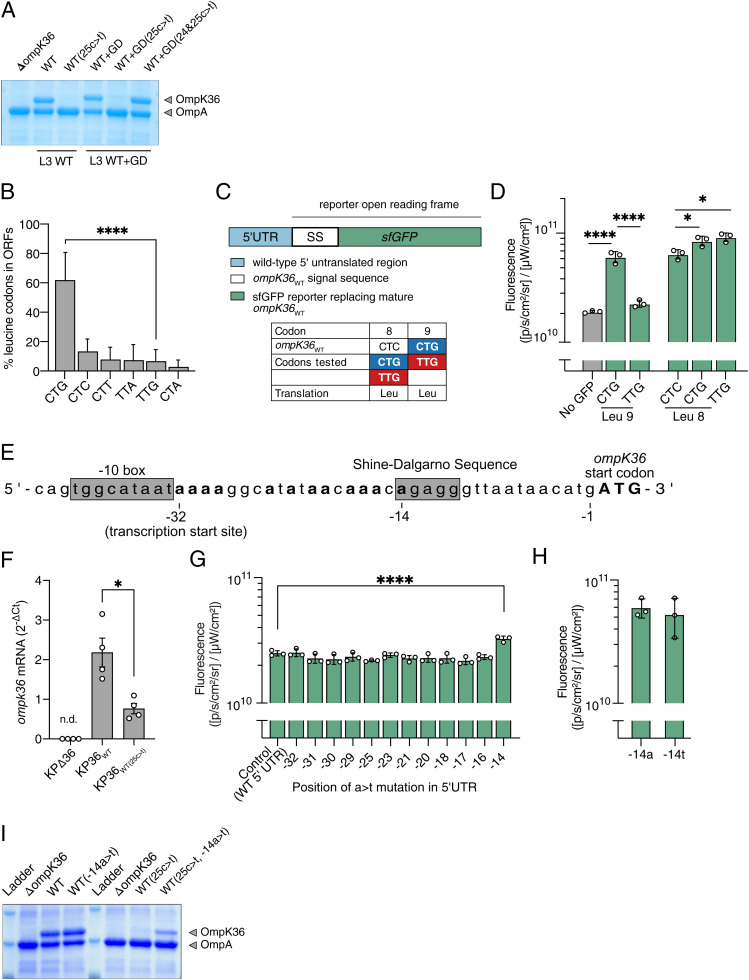
The 25c > t mutation is alleviated by a −14a > t substitution. (*A*) Representative Coomassie gel image of OM preparations demonstrating the reduced OmpK36 expression conferred by 25c > t in both WT and WT+GD *ompK36* backgrounds. The additional 24c > t in WT+GD(24&25c > t) reverses the OM depletion of OmpK36 in WT+GD(25c > t). (*B*) Proportion of leucine residues encoded by each of the six codons across individual ORFs in the ICC8001 *K. pneumoniae* genome. A significant difference was observed in the use of CTG relative to TTG; significance was determined by a paired t-test; ****P < 0.0001. (*C*) Schematic of the sfGFP reporter. The sequence encoding the mature OmpK36 protein was replaced with sfGFP, generating a chimeric fusion between the *ompK36*_WT_ SS (Leu9 CTG) and sfGFP. The inset table describes the mutations that were introduced. (*D*) Mutation of Leu9 CTG (*ompK36*_WT_) to TTG (*ompK36*_WT(25c > t)_) decreases the expression of the sfGFP reporter. Mutations in Leu8 (CTC codon in *ompK36*_WT_) to CTG or TTG codons lead to similarly increased levels of reporter expression. Fluorescence was determined using IVIS SpectrumCT. Graphs show the means of 3 biological repeats ± SD. Statistical significance was determined by one-way ANOVA with Tukey’s multiple comparison test. For multiple comparisons, only significant changes are indicated in the figure; *P < 0.05; ****P < 0.0001. All other comparisons were nonsignificant (*P* > 0.05). (*E*) Sequence of the *ompK36* 5′UTR. Adenine bases in the 5′UTR (in bold) were individually mutated to thymine. (*F*) RT-qPCR analysis of *ompK36* shows decreased transcript levels in the strain encoding *ompK36*_WT(25c > t)_ compared to *ompK36*_WT_. Bar charts show the mean ± SEM of 4 biological repeats. n.d., not detected. *P < 0.05 by paired Student’s *t* test. (*G*) Expression of the sfGFP reporter encoding the *ompK36*_WT(25c > t)_ SS is specifically increased upon mutation of the adenine in position −14 to thymine (−14a > t). The −14a marks the start of the SDS. Fluorescence was determined using the IVIS SpectrumCT. Graphs show the means ± SD of 3 biological repeats. Statistical analysis was performed by one-way ANOVA with Dunnett’s multiple comparison posttest. ****P < 0.0001. (*H*) Mutation of the adenine in position −14 to thymine (−14a > t) in the reporter construct encoding the *ompK36*_WT_ SS (*B*) does not impact sfGFP expression. Bar charts show the mean ± SD of 3 biological repeats. (*I*) Representative Coomassie gel image of OM preparations from isogenic *K. pneumoniae* strains with the indicated *ompK36* mutations in positions −14 (5′UTR) and 25 (ORF SS). Mutation of the adenine in position −14 to thymine (−14a > t) restores the expression of OmpK36 in the presence of the 25c > t mutation.

We next aimed to understand how the 25c > t mutation mediates an increase in meropenem resistance and an associated decrease in virulence. Since the mutation results in intermediate carbapenem resistance and virulence phenotypes, between those observed in *ompK36*_WT_ and Δ*ompK36*, we hypothesized that the changes were due to reduced OmpK36 abundance in the OM. Indeed, an analysis of isolated OM fractions by SDS-PAGE and Coomassie staining revealed that the strain encoding *ompK36*_WT(25c > t)_ exhibited a substantially reduced abundance of OmpK36 compared to that with *ompK36*_WT_ (Fig. 4*A*). In line with the meropenem MIC results, we also found that this pattern is reversed in the strain encoding *ompK36*_WT+GD(24&25c > t)_, which exhibits OmpK36 levels similar to the strain encoding *ompK36*_WT_ (Fig. 4*A*). Also, consistent with the meropenem MIC results, when the single 24c > t mutation was introduced into *ompK36*_WT_ or *ompK36*_WT+GD_, it did not have an observable impact on the abundance of OmpK36 in the OM (*SI Appendix*, Fig. S1).

### Reduced OmpK36 Abundance via the 25c > t Mutation Occurs Independently of Codon Bias.

We sought to elucidate the molecular mechanism linking the synonymous 25c > t mutation with the decreased OmpK36 OM abundance. We first explored whether reduced OmpK36 abundance is due to a decreased translation rate, resulting from a change in the codon from the commonly used CTG to the rarely used TTG at amino acid position 9 (Leu9). Overall, 64.9% of leucine residues in the ICC8001 genome are encoded by CTG and 5.9% are encoded by TTG (Fig. 4*B*); inspection of all the ST258/512 genomes confirmed a similar bias (CTG range: 57.0 and 65.1%; TTG range: 5.9 and 9.3%).

To investigate this, we generated a fluorescent ICC8001 reporter, in which the *ompK36* ORF was replaced with the *ompK36* SS fused to sfGFP (Fig. 4*C*). This reporter maintains the upstream promoter and regulatory regions at the monocistronic *ompK36* locus. Using sfGFP fluorescence as a proxy for protein expression, we found that the SS containing the 25c > t mutation (TTG codon) significantly reduced expression compared to that with the WT sequence (CTG codon) (Fig. 4*D*). This finding is consistent with the reduced OmpK36 abundance observed in the strain encoding *ompK36*_WT(25c > t)_ (Fig. 4*A*). We then used the CTC leucine codon located at amino acid position eight (Leu8) to test the impact of codon usage on expression. We generated synonymous mutants where Leu8 was encoded by either of the alternative CTG (common) or TTG (rare) codons. Both Leu8 CTG and TTG codons resulted in similar sfGFP expression, which was significantly higher than with the CTC codon found in *ompK36*_WT_ (Fig. 4*D*). This suggested that the underlying mechanism reducing OmpK36 abundance via the 25c > t mutation is not related to codon usage limiting the efficiency of translation elongation.

### Reduced OmpK36 Abundance Is Mediated by an Interaction between the ORF and 5′UTR.

Mutagenesis studies in *Escherichia coli* have suggested that specific intramolecular RNA interactions between nucleotides in the 5′ end of an ORF and the upstream 5′ untranslated region (UTR) can result in secondary structures that occlude the SDS, blocking ribosomal access (25, 26). This disrupts the initiation of translation, reducing protein expression, and in turn results in mRNA degradation as transcription and translation are tightly coupled (26). However, mutations reducing translation efficiency have not been identified as a naturally occurring mechanism tuning protein expression in adaptation to a host or environmental pressure. Nonetheless, we hypothesized that the reduced abundance of OmpK36_WT(25c > t)_ may be due to an inhibitory mRNA secondary structure, mediated by a specific a/u base pairing occurring between an adenine within the 5′UTR of *ompK36* (Fig. 4*E*) and the uracil encoded by 25t. We started investigating this by confirming the lower abundance of *ompK36* mRNA transcripts in the 25c > t mutant compared to WT by qRT-PCR (Fig. 4*F*), a finding consistent with increased mRNA degradation. We then individually mutated each adenine to a thymine (starting from the end of the −10 box/transcription start site) in the 5′UTR of the sfGFP reporter containing the 25c > t mutation in the SS. We hypothesized that the disruption of any base interaction between 25u and a position in the 5′UTR would be evidenced by an increased sfGFP expression. No significant changes in fluorescence were observed in adenine substitutions up to and including the −16 position (Fig. 4*F*). However, substitution of −14a, which marks the start of the SDS, abolished the effect of 25c > t on sfGFP signal suppression, with significantly increased fluorescence in the strain encoding the 14a > t mutation (*P* < 0.001). In order to exclude the possibility that −14a > t had a nonspecific effect on the functionality of the SDS due to enhanced ribosomal binding, we introduced −14a > t in the WT reporter (without 25c > t). Similar signal levels were observed in both reporters (−14a/25c and −14t/25c), suggesting that −14a > t alone does not affect protein expression (Fig. 4*H*).

We next investigated the effect of the −14a > t mutation on OmpK36 abundance in the context of *ompK36*_WT_ and *ompK36*_WT(25c > t)_. As expected, no effect on abundance was observed when the mutation was introduced into *ompK36*_WT_ (Fig. 4*I*). In contrast, introduction of the −14a > t mutation in the 5′UTR of *ompK36*_WT(25c > t)_ increased OmpK36 abundance (Fig. 4*I*). These findings suggest that the 25c > t mutation may result in an inhibitory mRNA secondary structure via a specific interaction with position −14 in the 5′UTR.

### The 25c > t Mutation Induces an mRNA Stem Loop Structure involving the SDS.

**Fig. 5.**
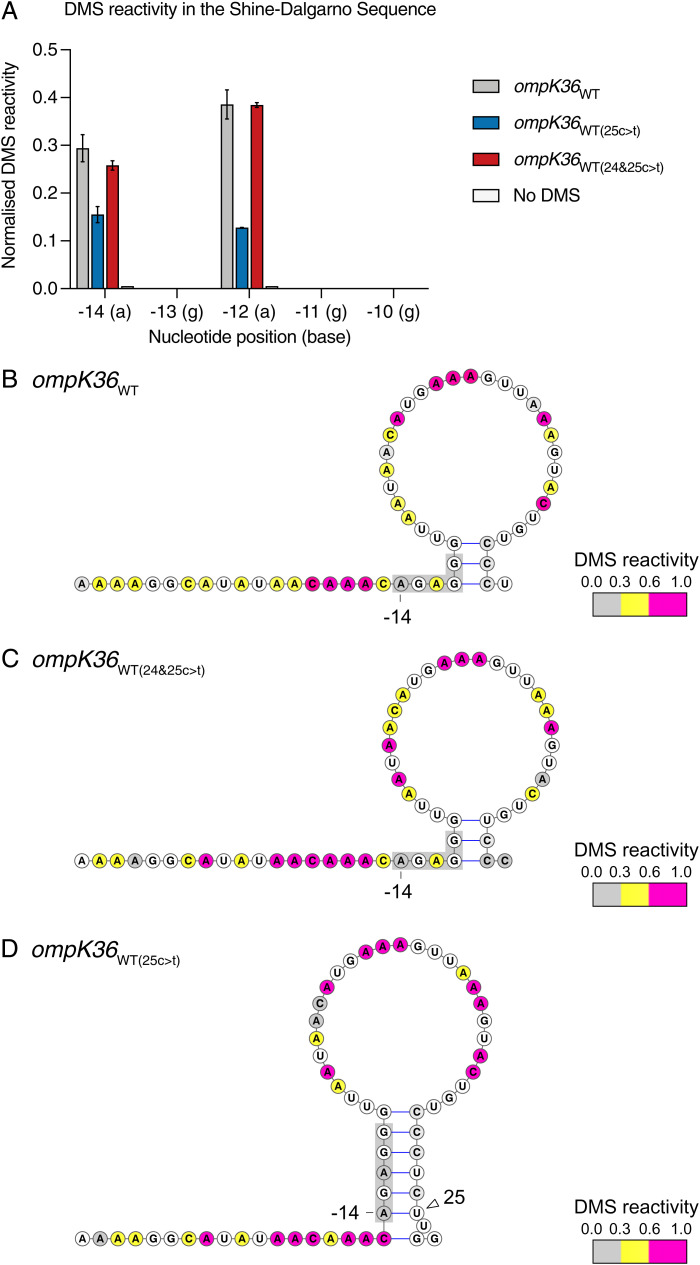
Position 25 in the *ompK36*_WT(25c > t)_ RNA induces a stem involving the SDS. (*A*) Side-by-side normalized DMS signal per nucleotide in the SDS from full-length *in vitro*–transcribed and refolded *ompK36*_WT_, *ompK36*_WT(25c > t)_, *ompk36*_WT(24&25c > t)_, and DMS-untreated *ompK36*_WT_. Higher values correspond to increased base accessibility. DMS signal (± SD) of 2 biological repeats for nucleotides −14a through −10g are shown. (*B*–*D*). DMS-constrained structure models of the 5′ end of *ompK36*_WT_ (*B*), *ompK36*_WT(24&25c > t)_ (*C*), and *ompK36*_WT(25c > t)_ (*D*). Nucleotides are colored by normalized DMS signal. The SDS in RNA structures are highlighted in gray. Arrows indicate the −14 position and the 25c > t position in *ompK36*_WT(25c > t)_.

To test the hypothesis that the 25c > t mutation results in an mRNA interaction between −14a and 25u that obstructs the SDS and prevents ribosome recruitment, we probed the structures of the *ompK36*_WT_ and *ompK36*_WT(25c > t)_ mRNAs using dimethyl sulfate (DMS) mutational profiling with sequencing (DMS-MaPseq) (27). This technique uses DMS modifications of adenine and cytosine bases in the RNA, detected as mutations during reverse transcription, to infer the accessibility of individual bases and subsequently build a model of the RNA structure. We found that adenines within the SDS are more accessible to DMS in *ompK36*_WT_ mRNA than *ompK36*_WT(25c > t)_ mRNA both in vitro and in vivo (Fig. 5*A*; *SI Appendix*, Fig. S2*A*). Introducing the 24c > t mutation in addition to 25c > t reversed the DMS accessibility of the SDS to that resembling *ompK36*_WT_ (Fig. 5*A* and Fig. S2*A*). As a negative control, we also tested a sample with no DMS treatment, which indeed showed minimal background signal (Fig. 5*A*; *SI Appendix*, Fig. S2*A*).

DMS-driven structure models showed visually that the SDS is largely accessible for both the WT and the double mutant (Fig. 5*B* and *C*; *SI Appendix*, Fig. S2*B*). In contrast, the SDS in *ompK36*_WT(25c > t)_ is sequestered into a stem structure, which incorporates the paired positions of −14 and 25 (Fig. 5*D* and Fig. S2*C*). These results provide direct evidence that the RNA structure occluding the SDS underlies the reduced expression of OmpK36 in strains encoding the 25c > t mutation. Altogether, these findings provide the precise molecular mechanism linking the occurrence of synonymous SNPs to altered OmpK36 abundance and meropenem resistance levels.

## Discussion

OmpK36 is a key porin in *K. pneumoniae*, reflected by its typically high abundance in the OM. However, its role in facilitating antibiotic entry has exerted an evolutionary pressure favoring mutations that restrict this process (2). Using large clinical genome collections, we identified a single, recurrently emerging, synonymous base substitution at the 25th nucleotide position of *ompK36* that increases carbapenem resistance by reducing OmpK36 expression. We observed that this 25c > t mutation usually occurs in the absence of L3 insertions, unless a 24c > t mutation that reverses the phenotype is also present, demonstrating that *K. pneumoniae* typically uses either OmpK36 pore constriction (via L3 insertions) or reduced OmpK36 abundance (via the 25c > t mutation) as a means for increasing resistance. Mechanistically, we show the significant impact a single synonymous substitution can have on protein expression, through the induction of a dramatic mRNA conformational change. We have shown that an adaptive mutation that restricts translation initiation can occur naturally.

In unraveling the molecular mechanism, our in vitro mutagenesis experiment showed that reduced OmpK36 expression via the 25c > t mutation is the result of a specific interaction between 25u in the mRNA and the first adenine base at the upstream SDS (−14a). The interaction was confirmed by solving mRNA structures with DMS-MaPseq. This technique revealed large conformational differences in the structures of the *ompK36*_WT_ and *ompK36*_WT(25c > t)_ transcripts, including the presence of a stem loop structure sequestering the SDS only in the *ompK36*_WT(25c > t)_. We propose that this stem loop structure, triggered by the interaction between 25u and −14a, impedes ribosomal binding and thereby reduces translation efficiency. Further evidence for this proposed mechanism also came from the mRNA structure of *ompK36*_WT(24&25c > t)_ (these double mutations also having been observed among clinical isolates), which shares structural homology with *ompK36*_WT_. This could explain the reversal of the phenotypes observed in this mutant compared with that encoding only 25c > t.

A change in mRNA conformation has previously been proposed to account for the increased growth rate of a *S. enterica* serovar Typhimurium strain, as mediated by an 5′ terminal synonymous SNP in the *proA* gene that evolved under specific laboratory conditions (15). Moreover, previous studies have demonstrated that experimentally induced mRNA secondary structures near the SDS decrease protein levels using a synthetic library of synonymous GFP variants (25) and, more recently, using two endogenous genes in *E. coli* (26). Crucially, however, the latter study proposed that the base composition at the start of genes has evolved to minimize the formation of these structures, as evidenced by a genome-wide analysis in *E. coli* (26). Thus, our study extends upon previous work by demonstrating that inhibitory mRNA secondary structures at the start of genes can be selected for during adaptation and indeed are used as a mechanism of regulation among naturally occurring and clinically relevant populations. More generally, our findings also add to the growing number of studies that show diverse mechanisms by which synonymous SNPs can affect protein expression (15, 17, 18). Future experiments will attempt to resolve whether the synonymous mutations in the SDS identified here directly affect ribosome binding and initiation complex formation (e.g., via toe-printing assays) and the effects this may have on mRNA stability (in addition to the expected impact on translation efficiency).

In keeping with the key role of OmpK36 in maintaining *K. pneumoniae* physiology, we demonstrated that the 25c > t mutation decreased bacterial replication in a murine pneumonia model. This correlated with reduced levels of serum IFNγ that plays an important role in *K. pneumoniae* lung infection in mice (28, 29). Our phylogenetic analyses suggested that this level of attenuation may be enough to impact transmission, with most isolates possessing this mutation forming singletons or pairs in the ST258/512 phylogeny (i.e., not being part of larger clonal expansions). Moreover, despite its frequent emergence, the total prevalence of the 25c > t mutation among the ST258/512 genome collection remained at 1.2%. This is similar to the prevalence of loss-of-function mutations in *ompK36* (0.7%) but is in sharp contrast to L3 insertions, which have spread widely via large clonal expansions to reach a prevalence of 47.0%. Nevertheless, despite the limited clonal expansion of 25c > t, our identification of this mutation in clinical isolates demonstrates that it does not preclude infection in patients. It is notable that the 24c > t appears to partially alleviate 25c > t–induced depletion in OM OmpK36 and is restricted to L3 insertion–containing *ompK36* variants. The latter modification significantly increases the meropenem MIC but is not accompanied with a significant fitness cost (5), in contrast to the 25c > t that achieves resistance at the cost of virulence. We therefore propose that the 24c > t is an epistatic mutation occurring within the *ompK36* ORF. While we are unable to unambiguously prove the evolutionary order of events leading to the genotype with the combined 24&25c > t mutations and L3 insertion, we suggest that 1) an isolate would acquire the 25c > t mutation (increasing meropenem MIC but reducing in vivo fitness) and then 2) an L3 insertion (further increasing meropenem MIC to above the requirement by *K. pneumoniae*) followed by 3) a 24c > t mutation (maintaining sufficiently high meropenem MIC but alleviating the fitness cost resulting from the 25c > t mutation).

Using our translational model of pneumonia, we could further show that the increased resistance conferred by the 25c > t mutation was enough to result in a clinical impact, thereby explaining its recurrent emergence (despite the fitness cost). Mice infected with *K. pneumoniae* expressing *ompK36*_WT_+KPC-2 could be successfully treated with meropenem, while those infected with *K. pneumoniae* possessing *ompK36*_WT(25c > t)_+KPC-2 could not. We therefore propose that the repeated emergence of 25c > t is driven by antibiotics, possibly due to prolonged exposure and/or subtherapeutic dosing; both are commonly encountered among critically ill patients, in whom antibiotic pharmacokinetics are difficult to predict at an individual patient level (30). Indeed, porin modifications occurring within the time course of a single infection have been reported previously (31, 32), demonstrating the potential for their emergence and selection given strong antibiotic pressure. Increased host susceptibility due to comorbidity and impaired immune responses in the population most at risk for *K. pneumoniae* disease could also render the associated fitness cost of 25c > t less significant.

While the frequency of 25c > t has remained low, we propose that its ongoing de novo emergence, together with the spectrum of other known OmpK36 modifications, impose a significant impact on patient treatment. Moreover, porin mutations that reduce carbapenem entry are not restricted to *K. pneumoniae* and are found in other WHO critical priority organisms such as *Pseudomonas aeruginosa* and *E. coli* (3, 33). This highlights the need for the development of effective antibiotic therapies that circumvent the reliance on diffusion through porins. While this has been achieved via the newly licensed cephalosporin cefiderocol, which instead enters via siderophore uptake systems (34), additional drugs are still needed.

In summary, our combined genomic, experimental, and translational approaches have uncovered a mechanism underpinning carbapenem resistance, mediated by synonymous mutations that alter the *ompK36* mRNA secondary structure. The associated dynamics of emergence and the expansion of these mutations are at the heart of an evolutionary conflict balancing resistance and virulence requirements in *K. pneumoniae*. We propose a central role for combining genomic surveillance with in vitro and translational evaluation to further our understanding of resistance mutations and to design and target our clinical interventions accordingly.

## Materials and Methods

### Genome Collections.

We used two genome collections to characterize the diversity and phylogenetic distribution of *ompK36* (and *ompK35*) variants. The first comprises 1,450 public *K. pneumoniae* genomes belonging to STs 258 and 512 and other nested STs, together with curated metadata obtained from associated publications (*SI Appendix*, Dataset S1). We obtained raw sequence reads if available (1,340 isolates) and short-read assemblies from the remainder (110 isolates). Assemblies were generated for all isolates with available raw sequence data using SPAdes v3.9.0 (35) and annotated with Prokka v1.14.5 (36). Kleborate v1.0.0 (37) was used for confirming the ST of each genome and determining the resistance gene content.

The second collection comprises a public genome collection of 16,086 *K. pneumoniae* available in Pathogenwatch (as of 28 February 2022) together with available metadata and genotyping data (38) (https://pathogen.watch/genomes/all?genusId=570&speciesId=573) (*SI Appendix*, Dataset S2).

### Phylogenetic Analysis of the ST258/512 Collection.

To generate a phylogenetic tree of the ST258/512 collection, we first used the “to_perfect_reads” function within Fastaq v3.17.0 (https://github.com/sanger-pathogens/Fastaq) to generate pseudosequence reads for isolates where only an assembly was available. We then mapped all sequence reads to the reference genome NJST258_1 (accession CP006923) (39), using Burrows Wheeler Aligner v0.7.17 (40). A pipeline comprising SAMtools mpileup v0.1.19 (40) and BCFtools v0.1.19 was used to call SNPs and generate a pseudogenome alignment. Gubbins v2.4.1 (41) was used to remove recombined regions from the alignment and generate a maximum likelihood tree with the remaining variable positions. The phylogenetic tree was rooted using an outgroup isolate from a closely related ST, ST895 (accession SRR5385992), which was later removed from the tree.

### Identification and Characterization of *ompK35 and ompK36* Genes.

The *ompK35 and ompK36* genes were identified in all short-read assemblies by performing BLASTn v2.6.0 (42) with a query gene from the reference genome ATCC43816 (the parental strain of ICC8001 (5) (accession CP009208). We required a single hit of each gene per genome that matched ≥10% of the query length, possessed ≥90% nucleotide similarity, and contained a start codon in order to unambiguously identify the gene. Nucleotide sequences were translated to protein sequences in Seaview v4.7 (43) using the standard genetic code, and the protein lengths were determined. Protein sequences were predicted to be intact if they contained an SS, an L3 sequence, and 16 beta-barrel sequences, as determined using BLASTx v2.6.0 (44). Intact protein sequences of each porin were aligned using MUSCLE v3.8 (45), and the different variants present were identified, taking into account all amino acid substitutions, insertions, and deletions.

### Analysis of *ompK36* Variation.

The alignment of intact *ompK36* nucleotide sequences from the ST258/512 collection was curated manually to ensure accurate positioning of bases around the L3 region (some of which were misaligned due to indels). We then used the sequences from the curated alignment to infer the likely base harbored by each internal node of the ST258/512 phylogenetic tree at each position in *ompK36*. This ancestral reconstruction was performed with PastML v1.9.30 (46) using maximum parsimony with the accelerated transformation (ACCTRAN) model. Using the predicted states of all internal nodes and the known bases in all terminal nodes, we determined the total number of changes at each position that had occurred across the ST258/512 tree.

### Codon Usage.

The frequency of different leucine codons was determined across all protein-coding genes in the ATCC43816 reference genome, as well as all annotated assemblies in the ST258/512 collection, using the EMBOSS v6.6.0.0 “cusp” tool (http://emboss.sourceforge.net/apps/release/6.3/emboss/apps/cusp.html).

### 5′UTR Analysis.

The 500-bp upstream region of *ompK36* was analyzed and annotated in Fig. 4*D* using BPROM (47). The SDS was identified manually by consensus with other *K. pneumoniae* genes.

### Data Analysis and Visualization.

Metadata, including *ompK36* variation, were mapped onto a phylogenetic tree of the ST258/512 collection using iTOL v5.7 (48) for use in figures. An interactive visualization of this genome collection can also be accessed using Microreact v157 (49) (https://microreact.org/project/1vWbaqARPRNc55n4yfdLyQ-ompk36).

Graph generation and statistical analysis were carried out in GraphPad Prism v9.0.0 for Mac (GraphPad Software; www.graphpad.com). Data were analyzed for normal distribution (based on D’Agostino–Pearson or Shapiro–Wilk normality tests) and if not normally distributed, a base 10 logarithmic transformation was applied before statistical analysis. When normality was not achieved by transformation, a nonparametric test was applied. Statistical tests applied for each analysis are described in associated figure legends.

Custom images of mice in Fig. 3 were made with Biorender. Images were edited and compiled into figures in Affinity Designer v1.8.4. (Serif Europe Ltd).

## Supplementary Material

Supplementary File

Supplementary File

Supplementary File

## Data Availability

Raw sequence data or assemblies from the extended ST258/512 collection were published in multiple manuscripts and obtained from the European Nucleotide Archive (ENA). Genome assemblies from the larger *K. pneumoniae* collection were obtained from Pathogenwatch (https://pathogen.watch/genomes/all?genusId=570&speciesId=573). A full list of accession numbers for all data are provided in Dataset S1 and S2; these are publicly available for download from the ENA website https://www.ebi.ac.uk/ena/browser/. Detailed materials and methods, including details on the generation of the strains in Tables 1 and 2, animal work, and the determination of the RNA structures using DMS-MaPseq are in *SI Appendix*, Materials and Methods. All study data are included in the article and/or supporting information.
