# Supplementary material for: Recurrent emergence of Klebsiella pneumoniae carbapenem resistance mediated by an inhibitory ompK36 mRNA secondary structure
Source: Proc Natl Acad Sci U S A. 2022 Sep 12;119(38):e2203593119. doi: 10.1073/pnas.2203593119 (PMC9499542; doi:10.1073/pnas.2203593119)
Supplement: Supplementary File [file pnas.2203593119.sapp.pdf]

Supporting Information Appendix

Recurrent emergence of *Klebsiella pneumoniae* carbapenem resistance mediated by an inhibitory *ompK36* mRNA secondary structure

Authors: Joshua L. C. Wong*, Sophia David*, Julia Sanchez-Garrido, Jia Z. Woo, Wen Wen Low, Fabio Morecchiato, Tommaso Giani, Gian Maria Rossolini, Konstantinos Beis, Stephen J. Brett, Abigail Clements, David M Aanensen, Silvi Rouskin and Gad Frankel

*These authors contributed equally to this work.

This file includes:

Materials and Methods

Figures S1 and S2

Supplementary Legends for Dataset S1 and S2

SI References

Materials and Methods

General cloning, molecular biology and strain generation

All *in vitro* assays and animal infections with KP were carried out using ICC8001, a strain derived by animal passage from ATCC43816(1).

A homologous recombination technique resulting in scarless markerless mutants was used to generate all strains based on the Standard European Vector Architecture platform pSEVA612S (JX560380.2) mutagenesis plasmid. Briefly, mutants were generated in two sequential recombination steps. The first step integrates the mutagenesis plasmid into the genome generating a merodiploid. The mutagenic plasmid was introduced by three part conjugation and homologous recombination promoted by lambda-recombinase expressed from a helper plasmid. The second step induces double strand DNA breaks by induction (L-arabinose) of I-SceI expression from the helper plasmid. Double stranded breaks are induced at I-SceI sites located on the chromosomally integrated mutagenesis vector, flanking the final integration region. This technique has been used to efficiently recombineer in KP(1) and other *Enterobacteriaceae*(2) as described in respective manuscripts.

Mutagenesis vectors were generated by routine molecular biological techniques. These included plasmid preparation (Monarch Plasmid Miniprep Kit, NEB), genomic DNA extraction from bacterial cells (DNEasy, QIAGEN), polymerase chain reaction (Q5 High-Fidelity 2x Master Mix and OneTaq, Quick-Load 2X Master Mix, both NEB), PCR clean-up (Monarch PCR and DNA Cleanup Kit, NEB) and gel extraction (Monarch Gel Extraction Kit, NEB), site directed mutagenesis (KLD enzyme blend, NEB) and Gibson assembly (Gibson Assembly Master Mix, NEB). Sanger sequencing was carried out to check vector construction and seamless integration into the genome (Eurofins Genomics). Custom primers in this study were made by Sigma-Aldrich.

ompK35 mutagenesis

ompK35 wild-type ORF (+500bp flanking regions) was cloned from ICC8001 genomic DNA with primers (P) 1/P2 and ligated by Gibson Assembly into pSEVA612S, linearised with P3/P4. The ORF was removed by inverse PCR to generate the genomic deletion vector (P5/P6) and plasmid recircularized from PCR product (KLD enzyme blend, NEB).

ompK36 mutagenesis

ICC8001**Δ***ompK36* was generated in previous work (Vector 5 in Wong et al., 2019). *ompK36* wild-type ORF (+500bp flanking regions) was cloned from ICC8001 genomic DNA with primers P7/P8 and ligated by Gibson assembly into pSEVA612S, linearised with P3/P4. In screening colonies by Sanger Sequencing we found the 25c>t mutation and this was not generated by site directed mutagenesis.

sfGFP vectors and -14a>t vectors

Leucine 9 CTG-sfGFP (wild-type) and Leucine 9 TTG-sfGFP (25c>t transition). The wild-type and 25c>t Sec signal sequences were cloned from the *ompK36* vectors generated above with P9/P10. sfGFP was amplified from our previously published glmS site insertion vector (Vector 13 in Wong et al., 2019) with P11/P12 and chimeric fusion generated in the mutagenesis vector ligated by Gibson Assembly.

Leucine 8 CTG-sfGFP and Leucine 8 TTG-sfGFP were generated by site-directed mutagenesis using P13/P14 and P15/P16 respectively using the Leucine 9 CTG-sfGFP (wild-type) as the template.

5' untranslated region mutagenesis of Leucine 9 TTG-sfGFP (25c>t transition). Mutants were generated by site-directed mutagenesis as follows: -32(a>t) (P17/P18), -31(a>t) (P17/P19), -30(a>t) (P17/P20), -29(a>t) (P17/P21), -25(a>t) (P22/P23), -23(a>t) (P22/P24), -21(a>t) (P22/P25), -20(a>t) (P22/P26), -18(a>t) (P22/P27), -17(a>t) (P22/P28), -16(a>t) (P22/P29), -14 a>t(a>t) (P22/P30) using leucine 9 TTG sfGFP as the template. The same primer pair

(P22/P30) was used to introduce the -14a>t mutation into Leucine 8 CTG-sfGFP, *ompK36WT* and *ompK36WT(25c>t)* vectors.

The 24c>t mutation was introduced by SDM using primers P31 and P32.

Introduction of pKpQIL KPC-2 plasmid

We introduced pKpQIL KPC-2 by conjugation using an *E. coli* donor following our previously published protocol(1).

Outer membrane preparations and gel electrophoresis

Outer membrane proteins were purified according to a previously described protocol with several modifications(3). Saturated overnight cultures of bacteria were grown in LB (10g/L NaCl) and sonification was performed at 25% amplitude for 10 bursts of 15 seconds each. 10 µg of protein was separated by SDS-PAGE using 12% acrylamide Mini-protean TGX precast gels (Bio-Rad, USA). Gels were stained with Coomassie (Sigma-Aldrich) and imaged on a ChemiDoc XRS+ (Biorad, USA).

RNA isolation and reverse transcription quantitative PCR (RT-qPCR)

300 µl of an LB overnight culture of KP were treated with RNAprotect Bacteria reagent (Qiagen) and centrifuged at 5000 xg for 10 min. The bacterial pellet was digested in 100 µl TE buffer with 15 mg/ml Lysozyme (Sigma-Aldrich) and 20 µl Proteinase K (Qiagen) for 10 min according to manufacturer's guidelines. RNA was isolated using the RNeasy minikit (Qiagen) following the manufacturer’s instructions, and RNA concentration was determined using a Nanodrop 2000 spectrophotometer (ThermoFisher Scientific). 1 µg of RNA was treated with RNase-free DNase (Sigma-Aldrich) for 1 h at 37 °C and cDNA was then synthesized using a Moloney murine leukemia virus (MMLV) reverse transcription kit with random primers following the manufacturer´s protocol (Promega). To check for the presence of remaining DNA, a reaction mixture without the MMLV reverse transcriptase was also included (NRT). qPCR was performed using the Power Up SYBR Green master mix (ThermoFisher Scientific) and the

following primers 33/34 (*ompK36*) and 35/36 (*rpoB*). The assay was run on a StepOnePlus System (Applied Biosystems) and results were analysed using the StepOne software (Applied Biosystems). Relative gene expression levels were analyzed by using the $2^{-\Delta CT}$ (where C_T is threshold cycle) method.

<u>IVIS sfGFP fluorescence measurement</u>

Saturated overnight cultures of strains were diluted 1:2000 in PBS. 20ul of suspension was plated onto an agar plate and allowed to dry. Following overnight incubation (16 hours exactly) plates were removed and imaged on an IVIS Spectrum CT imaging platform (1 second exposure, Ex 465nm/Em520nm). Fluorescence over the area of the spot was quantified using Living Image Software v5.4.3 (Perkin Elmer).

<u>Meropenem Minimum Inhibitory Concentrations (MIC)</u>

The meropenem MIC of strains was assessed by broth microdilution by broth microdilution in biological triplicate according to the ISO 20776-1:2019 standard and the median value taken as the final MIC.

<u>Animal work</u>

8-10 week old, female, 18-20g BALB/c mice were purchased from Charles River, UK. All animal work took place in the institution animal facility (Association for Assessment and Accreditation of Laboratory Animal Care accredited) under the auspices of the Animals (Scientific Procedures) Act 1986 (PP7392693). Work was approved locally by the institutional ethics committee.

<u>Housing and Randomisation</u>

Upon arrival animals were independently randomized into cages of 5 animals and housed for an acclimatisation period of 1 week. Mice received food and water *ad libitum* and were housed in a 12hour/12hour light dark cycle. Identification of animals within groups was achieved by ear notching without anaesthesia at least 24 hours in advance of procedures.

Intratracheal administration of inoculum and infection

Anaesthesia was induced by intraperitoneal injection (BD Microlance 27G 13mm needle) of 80mg/kg ketamine and 0.8mg/kg medetomidine. Pre-intubation body temperature was maintained by contact with a heat mat (Harvard Apparatus, U.K) and eye lubricant (2.0mg/g carbomer, Alcon UK) was applied.

Intubation was achieved as previously described(1)by placement of a 21G catheter (21G IV peripheral catheter (Insyte) BD Medical) using a fibreoptic intubation set (Kent Scientific) following the enclosed instructions.

Inoculum (dose defined in graphs) was prepared by dilution of saturated overnight cultures into phosphate buffered saline (PBS) (total inoculum volume 50ul) and following inspiration it was dispersed with 2 x 200ul air flushes. An aliquot of inoculum was enumerated for each administration and all inoculums were ±10% of the stated figure.

Mice recovered at 32°C (Warm Air System, Safety Cabinet Version red, VetTech, UK) until spontaneous movement and received 0.8mg/kg subcutaneous (BD Microlance 27G 13mm needle) atipamezole to reverse the α-agonist into the neck scruff.

At the experimental end-point animals were anesthetized with ketamine 100mg/kg and 1mg/kg medetomidine by intraperitoneal injection (BD Microlance 27G 13mm needles). Following the induction of anaesthesia, blood was collected by a transdiaphragmatic inferior approach cardiac puncture (BD Microlance 25G 25mm needles) and animals were then humanely killed, under anaesthesia, by cervical dislocation.

An aliquot of blood (20ul) was taken and diluted into 180ul of hypotonic lysis solution (1mM EDTA in water) for enumeration of bacterial counts. The rest was placed into a microtainer (BD Microtainer for serum collection, BD Medical) and allowed to clot for 1 hour. This was then spun according to the manufacturer’s guidance and aliquoted into cryobanking tubes (Greiner Bio-One) and frozen at -80 °C until analysis.

Lungs were dissected out, weighed and then homogenised in gentlemacs C-tubes (Miltenyi Biotec) containing 3 ml complete RPMI media (RPMI supplemented with 10% heat inactivated FBS, 10 mM HEPES and 1mM Sodium Pyruvate) using a gentleMac Octo dissociator (Miltenyi Biotec) using the program m_lung_1 twice.

Bacteria were enumerated from the lung homogenate and blood by dilution in PBS and plating onto solidified LB agar containing 50ug/ml rifampicin (Merck, UK).

Antibiotic delivery

We combined the meropenem (100mg/kg) with cilastatin (100mg/kg) (Merck, UK), a renal dihydropeptidase inhibitor, to reduce the *in vivo* metabolism of meropenem as the murine enzyme isoform has increased activity against this carbapenem compared to human enzyme. Drugs were diluted in dH_2O. dH_2O alone was administered to vehicle control animals. Doses were delivered at 6 hourly intervals as previously employed in a murine carbapenemase producing KP model(4). The drug mixture was delivered by intraperitoneal infection (BD Microlance 27G 13mm needles) to animals at 6 hourly intervals and the iliac fossa used was alternated between doses.

Cytokine bead assay

Serum IFN-γ was assessed using a custom-made mouse panel 13-plex kit (LEGENDplex, BioLegend) following the manufacturer's instructions. Cytokine levels were acquired using a FACSCalibur flow cytometer (BD Biosciences), and analyses were performed using LEGENDplex data analysis software (BioLegend). A biological repeat was considered valid only if at least 80% of all the samples run were above the limit of detection; any values below the detection limit within a repeat such as this were assumed to be the lowest value detectable by the assay for statistical analysis. Values below the detection limit in graphs were displayed as 1 for visualisation.

Determination of RNA structures using DMS-MaPseq

In vitro DMS modification

Plasmids encoding the wildtype and mutant *ompk36* were isolated using miniprep (Zymo) and amplified by PCR using a forward primer containing the T7 promoter sequence (P37) and a reverse primer (P38). The PCR product was used for T7 Megascript *in vitro* transcription (ThermoFisher Scientific) according to manufacturer’s instructions. Next, the RNA was purified using RNA Clean and Concentrator™-5 (Zymo). 10µg RNA was denatured at 95°C for 1 min. Denatured RNA was refolded by incubating in 340mM sodium cacodylate buffer (Electron Microscopy Sciences) and 5mM $MgCl^{2+}$, such that the volume was 97.5µl, for 20 min at 37°C. Then, 0.5% DMS (Millipore-Sigma) was added and incubated for 4 mins at 37°C while shaking at 800 r.p.m. on a thermomixer. Subsequently, DMS was neutralized by adding 60µl β-mercaptoethanol (BME) (Millipore-Sigma). The DMS-modified RNA was purified using RNA Clean and Concentrator™-5 (Zymo) and eluted in 10µl water.

DMS-MaPseq library generation of whole-length in vitro DMS-modified RNA

500ng of RNA purified from previous steps was used as input. DMS-MaPseq libraries were prepared using IDT’s xGen™ Broad-Range RNA Library Prep Kit with slight modifications. Briefly, RNA was fragmented using manufacturer’s instructions for 2 minutes without adding reagent F2 (dNTPs). After 2 minutes, fragmentation mix was placed on ice immediately. The mixture was then added with the TGIRT reverse transcriptase mix, which consists of 1µl TGIRT, 1µl water, 1µl enzyme R1 (RNAse inhibitor) and 1µl DTT, and incubated at room temperature for 30 minutes. Then, F2 (dNTPs) was added and the fragmented RNA mixture was reverse transcribed under the conditions: 20°C for 10 mins, 42°C for 10 mins, 55°C for 60 mins, denaturation by adding 1µl 4M NaOH at 95°C for 3 mins. The mixture was neutralized by adding 2µl of 4M HCl and the volume of neutralized mixture brought up to 50µl. Then, the cDNA was cleaned up using 1x volume ratio of SPRI beads (Beckman Coulter) and eluted in 10µl EDTA TE. Samples were then adapted, extended, ligated and amplified for 10 cycles

following IDT's instructions. The libraries (~300-400 bp) were gel-purified on an 8% TBE polyacrylamide gel (ThermoFisher Scientific) and precipitated using isopropanol. The libraries were then loaded on iSeq-100 sequencing flow cell with iSeq-100 High-throughput sequencing kit and library was run on iSeq-100 (paired-end run, 151 x 151 cycles).

In vivo DMS modification, total RNA extraction and rRNA subtraction

For *in vivo* DMS modification, 500µl of exponentially growing *E. coli* were incubated with 10µl DMS for 3 mins at 37°C while shaking at 800 r.p.m. on a thermomixer. DMS was quenched by adding 500µl 30% BME, followed by 3 min 30% BME wash and 1x PBS wash. Then, bacterial pellets were resuspended and incubated at room temperature for 5 mins in 500µl RNAprotect® bacteria reagent (QIAGEN). Samples were centrifuged at 16000g for 5 mins and supernatant removed. Pellets were resuspended in 100µl of 15mg/ml lysozyme solution plus 20µl proteinase K and mixed by vortex for 10 seconds. Samples were incubated at room temperature for 10 mins, vortexing for 10 seconds every 2 mins. Next, samples were added with 350µl of buffer RLT plus BME (10µl BME for every 1ml of RLT), vortex to mix. Then, 250µl of 96-100% ethanol was added to samples. RNA was extracted using the RNeasy® Mini kit (QIAGEN) following manufacturer's instructions. DNA was digested from 5-10µg of total RNA per sample using the TURBO DNA-free kit (ThermoFisher Scientific) and purified using RNA Clean and Concentrator™-5 kit (Zymo). Following that, ribosomal RNAs were depleted using the Ribominus™ Transcriptome Isolation kit for bacteria (ThermoFisher Scientific) following manufacturer's instructions. RNA was purified using RNA Clean and Concentrator™-5 (Zymo) and eluted in 10µl water.

DMS-MaPseq library generation of target sequence-specific *in vivo* DMS-modified RNA

To reverse transcribe, rRNA-depleted total RNA purified from the previous steps was added to 4µl 5x FS buffer, 1µl dNTP, 1µl 0.1M DTT, 1µl RNase Out, 1µl 10uM reverse primer (P39) and 1µl TGIRT-III (Ingex). The reaction was incubated for 1.5h at 60°C. Then, to degrade the RNA, 1ul 4M NaOH was added and incubated for 3min at 95°C. The cDNA was purified in

10µl water using the Oligo Clean and Concentrator™ kit (Zymo). Next, 1µl of cDNA was amplified using Advantage HF 2 DNA polymerase (Takara) for 30 cycles according to the manufacturer's instructions (P40/P41). The PCR product was purified using E-Gel™ SizeSelect™ II 2% agarose gel (Invitrogen). RNA-seq library for 300bp insert size was constructed following the manufacturer's instructions (NEBNext Ultra™ II DNA Library Prep Kit). The library was loaded on iSeq-100 sequencing flow cell with iSeq-100 High-throughput sequencing kit and library was run on iSeq-100 (paired-end run, 151 x 151 cycles).

DMS-MaPseq analysis

To determine the DMS signal, FASTQ files were processed and analyzed using the DREEM (Detection of RNA folding Ensembles using Expectation-Maximization clustering) pipeline (5). Briefly, reads were trimmed using TrimGalore (github.com/FelixKrueger/TrimGalore) to remove Illumina adapters. Trimmed paired reads were then mapped to *ompk36*$_{WT}$ (accession number CP009208.1, annotated as ompC4), 25c>t and double mutant using Bowtie2 with the parameters: --local --no-unal --no-discordant --no-mixed -L 12 -X 1000. A bit vector was generated for each pair of aligned reads and their mutational signatures were analyzed using the DREEM algorithm(5). The ratio of mismatches and deletions to total coverage at each nucleotide position was calculated to quantify the population average DMS reactivity at each position. Then, DMS reactivities were normalized to the median of the top 5% of DMS reactivities to a scale of 0 to 1. The normalized DMS signals were used as folding constraints for predicting RNA secondary structures with the program RNAstructure v.6.0.1(6). RNA secondary structures were visualized using VARNA v.3.93 (7).

Primers used

P1 GCCAGTATAGGGATAACAGGGTAATCCGGTACCGCCCGGTGTCTG

P2 CGCCGGATTACCCTGTTATCCCTAGGAAATCACAATTATGTTACG

P3 ATTACCCTGTTATCCCTATAC

P4 TAGGGATAACAGGGTAATCCG

P5 TCTGCAGTACACCCCTTC

P6 CATTATTTATTACCCTC

P7 GATTACGCCGGATTACCCTGTTATCCCTAGGCCTAATTGATTGATTAATAG

P8 GGCCAGTATAGGGATAACAGGGTAATAGCCCCACAGGTTGACCAGC

P9 GTTGCAAGCTGCATAAC

P10 CGCATTTGCTGCGCCTGCTAC

P11 GTAGCAGGCGCAGCAAATGCGCGTAAAGGCGAAGAGCTG

P12 GTTATGCAGCTTGCAACCGCGAAGTAATCTTTTCG

P13 TTAACTTTCATGTTATTAACCCTCTGTTTGTTATATG

P14 AGTACTGTCCCTGCTGGTACCGG

P15 AGTACTGTCCTTGCTGGTACCGG

P16 TTAACTTTCATGTTATTAACCCTCTG

P17 GCATATAACAAACAGAGGGTTAATAAC

P18 CTTTAATTATGCCACTGC

P19 CTTATATTATGCCACTGC

P20 CTATTATTATGCCACTGC

P21 CATTTATTATGCCACTGC

P22 CTTTTATTATGCCACTGC

P23 GCTTATAACAAACAGAGGGTTAATAAC

P24 GCATTTAACAAACAGAGGGTTAATAAC

P25 GCATATTACAAACAGAGGGTTAATAAC

P26 GCATATATCAAACAGAGGGTTAATAAC

P27 GCATATAACTAACAGAGGGTTAATAAC

P28 GCATATAACATACAGAGGGTTAATAAC

P29 GCATATAACAATCAGAGGGTTAATAAC

P30 GCATATAACAAACTGAGGGTTAATAAC

P31 TACTGTCCCTTCTGGTACCGG

P32 CTTTAACTTTCATGTTATTAACCCTCTG

P33 CCAGACCTACAACGCAACT

P34 CGCTCCAGATCCTTACCTTTAG

P35 AAGGCGAATCCAGCTTGTTCAGC

P36 TGACGTTGCATGTTCGCACCCATCA

P37 TAATACGACTCACTATAGGAAAAGGCATATAACAAACAGAGGG

P38 AAGAGTATACCAGCGAGGTTAAACCGGAC

P39 CCTGAACGTTGTATTCCC

P40 GGAAAAGGCATATAAC

P41 CCTGAACGTTGTATTCCC

Supplementary Figures

Figure S1.

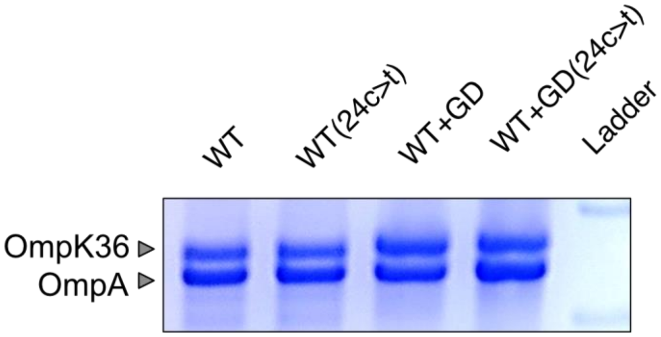

Representative Coomassie gel image of outer membrane (OM) preparations demonstrating no change in OmpK36 expression conferred by the introduction of a 24c>t mutation in in both WT and WT+GD *ompK36* backgrounds.

Figure S2.

A.

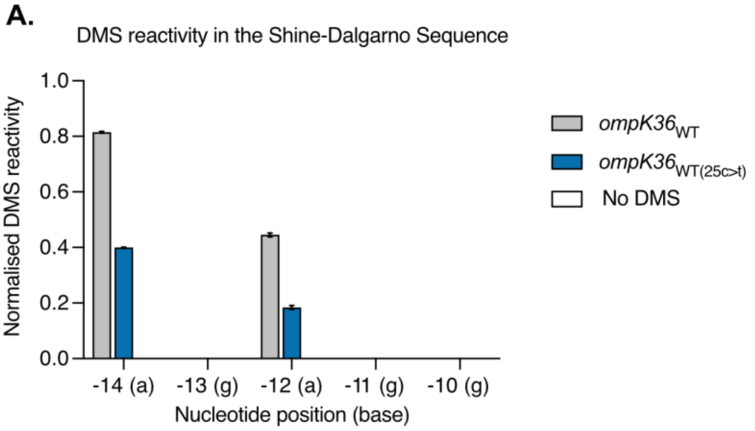

B. *ompK36*$_{WT}$

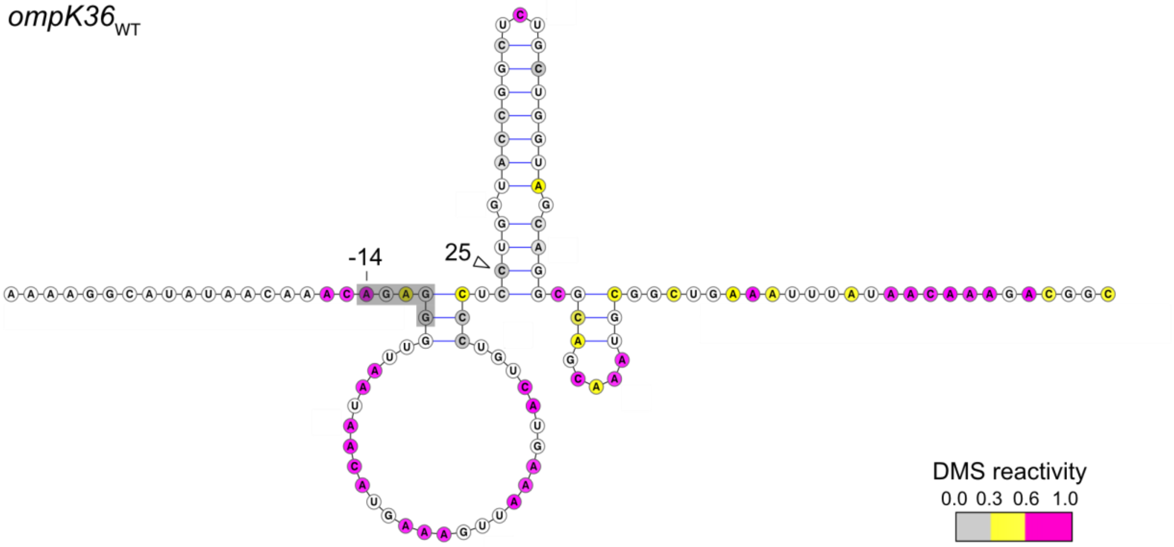

C. *ompK36*$_{WT(25c>t)}$

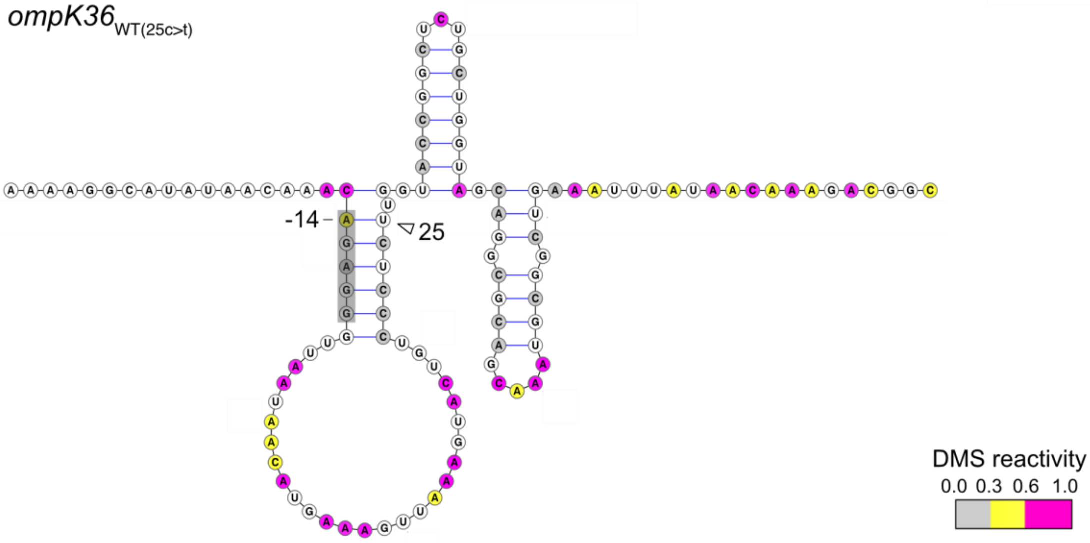

A. Side-by-side normalized dimethyl sulfate (DMS) signal per nucleotide in the Shine-Dalgarno sequence from full length *in vivo*-transcribed and refolded *ompK36*$_{WT}$, *ompK36*$_{WT(25c>t)}$ and DMS-untreated *ompK36*$_{WT}$. Higher values correspond to increased base accessibility. DMS signal (± SD) of 2 biological repeats for nt -14a through -10g are shown.

B&C. DMS-constrained structure models of the 5' end of *ompK36*$_{WT}$ **(B)** and *ompK36*$_{WT(25c>t)}$ (**C**) Nucleotides are coloured by normalised DMS signal. The SDS in RNA structures are highlighted in grey. Arrows indicate the -14 position and the 25c>t positions.

Representative Coomassie gel image of outer membrane (OM) preparations demonstrating no change in OmpK36 expression conferred by the introduction of a 24c>t mutation in in both WT and WT+GD *ompK36* backgrounds.

Supplementary Table Legends

Dataset S1. Metadata and genotyping data for a curated collection of 1450 KP ST258/512 genomes. See corresponding excel file.

Dataset S2. Metadata and genotyping data for 16,086 KP genomes available in Pathogenwatch. See corresponding excel file.

References

1. J. L. C. Wong, *et al.*, OmpK36-mediated Carbapenem resistance attenuates ST258 Klebsiella pneumoniae in vivo. *Nature Communications* **10**, 3957 (2019).
2. C. N. Berger, *et al.*, Citrobacter rodentium Subverts ATP Flux and Cholesterol Homeostasis in Intestinal Epithelial Cells In Vivo. *Cell Metabolism* **26**, 738-752.e6 (2017).
3. M. G. Wise, E. Horvath, K. Young, D. F. Sahm, K. M. Kazmierczak, Global survey of Klebsiella pneumoniae major porins from ertapenem non-susceptible isolates lacking carbapenemases. *Journal of Medical Microbiology* **67**, 289–295 (2018).
4. K. Ota, N. Kaku, K. Yanagihara, Efficacy of meropenem and amikacin combination therapy against carbapenemase-producing Klebsiella pneumoniae mouse model of pneumonia. *J Infect Chemother* **26**, 1237–1243 (2020).
5. P. Tomezsko, H. Swaminathan, S. Rouskin, Viral RNA structure analysis using DMS-MaPseq. *Methods* **183**, 68–75 (2020).
6. J. S. Reuter, D. H. Mathews, RNAstructure: software for RNA secondary structure prediction and analysis. *BMC Bioinformatics* **11** (2010).
7. K. Darty, A. Denise, Y. Ponty, VARNA: Interactive drawing and editing of the RNA secondary structure. *Bioinformatics* **25**, 1974–1975 (2009).
